# CGM-Based Glycemic Metrics Support Estimating Nutritional Risk After Total Pancreatectomy: An Exploratory Retrospective Study

**DOI:** 10.3390/jcm14197124

**Published:** 2025-10-09

**Authors:** Ryoma Nakamura, Miyuki Yanagimachi, Kento Mitsuhashi, Masato Yamaichi, Wataru Onodera, Atsufumi Matsumoto, Eri Sato, Yusuke Tando, Yukihiro Fujita

**Affiliations:** 1 Department of Endocrinology and Metabolism, Hirosaki University Graduate School of Medicine, Hirosaki 036-8562, Japan; 2 Department of Biomedical Laboratory Medicine, Hirosaki University Graduate School of Health Sciences, Hirosaki 036-8203, Japan

**Keywords:** total pancreatectomy, malnutrition, pancreatogenic diabetes mellitus, continuous glucose monitoring, glycemic fluctuation

## Abstract

**Introduction:** After total pancreatectomy, patients inevitably develop pancreatogenic diabetes with marked glycemic variability and high risk of malnutrition due to both endocrine and exocrine insufficiency. Weight loss and malnutrition can occur even in those with adequate dietary intake and plausible pancreatic enzyme replacement. We hypothesized that glycemic variability is associated with nutritional decline. **Methods:** We retrospectively analyzed 14 patients who underwent continuous glucose monitoring (CGM) after total pancreatectomy. Nutritional status was assessed using the Geriatric Nutritional Risk Index (GNRI), and patients were classified into malnutrition-risk progression or nutrition-maintaining groups. Then, we evaluated glycemic indices, dietary intake, anthropometry, and pancreatic enzyme replacement therapy (PERT). **Results:** Insulin use, PERT dose, and dietary intake were approximately comparable between groups. In contrast, the malnutrition-risk progression group showed significantly higher mean glucose and time above range, and lower time in range (TIR). Importantly, TIR consistently showed an inverse association with malnutrition-risk progression across models adjusted for clinical covariates, including time since pancreatectomy, primary diagnosis, insulin regimen, and pancrelipase dose. These findings indicate that the observed relationship between lower TIR and worsening GNRI was independent of dietary intake and adequacy of enzyme replacement therapy, underscoring TIR as a clinically meaningful indicator of nutritional decline in this population. **Conclusions:** Hyperglycemia and reduced TIR were significantly associated with worsening GNRI after total pancreatectomy, independent of dietary intake or PERT. CGM-based glycemic metrics may help identify patients at risk of malnutrition and guide postoperative management.

## 1. Introduction

Pancreatogenic diabetes mellitus or type 3C diabetes is caused by various pancreatic diseases, such as pancreatitis (acute or chronic), pancreatic trauma, pancreatic neoplasia, and hemochromatosis, as well as by pancreatectomy, including total pancreatectomy [1]. Total pancreatectomy results in the complete loss of pancreatic endocrine and exocrine functions. Loss of endocrine function leads to insulin-dependent diabetes mellitus, and exocrine insufficiency causes maldigestion and malabsorption of nutrients, mainly fat, in patients.

Total pancreatectomy leads to exocrine insufficiency, such as a lack of pancreatic enzyme secretion and impaired bicarbonate secretion. Impaired bicarbonate secretion also leads to fat maldigestion and malabsorption via impaired micelle formation by bile acids due to increased acidity in the duodenum [2]. As a result, maldigestion and malabsorption of macronutrients lead to weight loss, sarcopenia, and progressive malnutrition, while malabsorption of fat leads to clinical symptoms such as steatorrhea and abdominal symptoms [3].

Pancreatogenic diabetes is characterized by insulin and glucagon deficiencies. Impaired glucagon secretion causes impaired glyconeogenesis, resulting in severe hypoglycemia, prolonged hypoglycemia, and increased glycemic fluctuations during insulin therapy [4]. Appropriate pancreatic enzyme replacement and glucose management with insulin therapy are essential, as pancreatic hormones play a life-sustaining role and are indispensable not only for maintaining well-being but also for maintaining life.

One characteristic of pancreatogenic diabetes mellitus after total pancreatectomy is prolonged severe hypoglycemia, which makes it difficult to maintain good glycemic management. Severe hypoglycemia has been reported to cause death from pancreatogenic diabetes mellitus in Japan [5], and hypoglycemia is considered to play a role in the prognosis and quality of life of patients with pancreatogenic diabetes mellitus. The Japanese guidelines for diabetes treatment suggest that an HbA1c level < 8.0% should be set as the treatment goal for patients with comorbidities and functional disorders when it is difficult to intensify treatment [6].

It is crucial to examine short- and long-term glycemic management in patients with pancreatogenic diabetes mellitus. HbA1c accurately reflects 1–2 months of glycemic management, although it is incapable of evaluating daily glycemic fluctuations [7]. Other endpoints are required to evaluate high glucose fluctuations, which are often observed in patients with pancreatogenic diabetes mellitus. Recently, the usefulness of continuous glucose monitoring (CGM) for glycemic control in patients with diabetes has been demonstrated. An international meta-analysis comparing self-monitoring blood glucose (SMBG) and CGM reported that CGM was associated with a lower risk of hypoglycemia [8]. After 1 year of continuous intermittently scanned CGM in patients with type 1 diabetes, the mean glucose level significantly reduced, and the time in range (TIR) increased, improving HbA1c levels without increasing hypoglycemic exposure. A review of hypoglycemia prevention, including sensor-augmented pump therapy (SAP), CSII, and MDI, reported that only real-time CGM reduced both severe hypoglycemia and unawareness of hypoglycemia in patients with type 1 diabetes treated with multiple injections [9]. However, reports on the association between CGM and nutritional status are scarce.

Few reports have examined the impact of glycemic variability on the long-term nutritional status of patients with pancreatogenic diabetes mellitus. We hypothesized that glucose variability indices, particularly time in range (TIR) and time above range (TAR), are associated with nutritional risk assessed by the geriatric nutritional risk index (GNRI) in patients after total pancreatectomy. The primary endpoint of this study was the association between TIR/TAR and GNRI. Our principal finding was that CGM-based glycemic metrics may help identify patients at risk of malnutrition and guide postoperative management.

## 2. Materials and Methods

### 2.1. Patients

**Figure 1:**
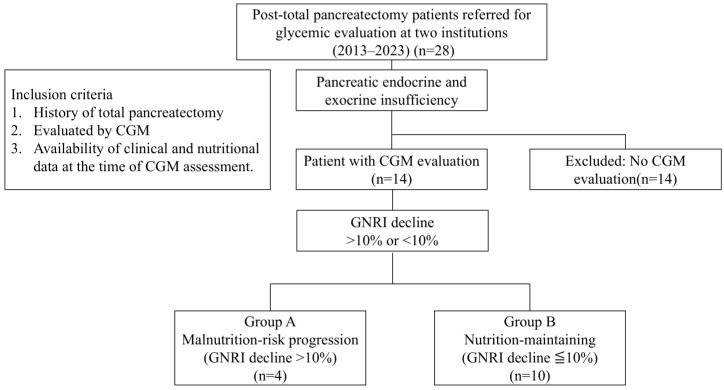
Study pipeline. Patients who underwent total pancreatectomy and were referred to our departments for glycemic evaluation between 2013 and 2023 were retrospectively reviewed. All included patients inevitably developed pancreatic endocrine and exocrine insufficiency. Patients without CGM evaluation were excluded from the analysis. Glycemic variability was assessed using CGM metrics (mean glucose, time in range [TIR], time above range [TAR], and time below range [TBR]). Nutritional status was evaluated based on the Geriatric Nutritional Risk Index (GNRI). Patients with a GNRI decline > 10% were classified as Group A (malnutrition-risk progression), while those with a decline < 10% were classified as Group B (nutrition-maintaining).

This retrospective observational study included patients who underwent total pancreatectomy and were referred for glycemic evaluation with CGM at the Departments of Diabetes, Endocrinology, and Metabolism, Hirosaki University Hospital, and Hachinohe City Hospital between January 2013 and August 2023. Inclusion criteria were: (1) history of total pancreatectomy, (2) CGM performed, and (3) availability of clinical and nutritional data at the time of CGM assessment. A study pipeline is shown in Figure 1. During the study period, a total of 28 patients who had undergone total pancreatectomy were referred to our departments for glycemic evaluation. Among them, we included 14 patients who underwent total pancreatectomy and subsequently underwent CGM (Guardian™ Sensor 3^®^ [Medtronic Japan Co., Ltd., Tokyo, Japan] or the FreeStyle Libre Flash Glucose Monitoring System^®^ [Abbott Japan LLC, Tokyo, Japan]) at Hirosaki University Hospital or Hachinohe City Hospital. Patients without a CGM evaluation were excluded. All included patients inevitably developed pancreatic endocrine and exocrine insufficiency. Nutritional status was evaluated using the GNRI. The study flow, from surgery to classification into Group A (malnutrition-risk progression) and Group B (nutrition-maintaining), is illustrated in Figure 1. An opt-out informed consent protocol was used for the use or collection of participant data for research purposes.

### 2.2. Nutritional Assessments

We collected nutritional indices (body mass index [BMI], serum albumin [Alb], and serum cholesterol) before and after surgery and evaluated the Geriatric Nutritional Risk Index (GNRI). The GNRI score is calculated as [14.89 × serum Alb (g/dL)] + [41.7 × (present body weight/ideal body weight/kg)] and is a predictor of the incidence of postoperative complications and mortality in older adults. The ideal body weight (WLo: via Lorentz formula) was calculated for men as: height − 100 − [(height − 150)/4]; for women as: height − 100 − [(height − 150)/2.5]. GNRI scores were classified as: ≥98, good nutritional evaluation; <98 and ≥92, low risk; <92 and ≥88, moderate risk; and <88, high risk [10].

We allocated patients to two groups, the malnutrition-risk progression group (Group A) and the nutrition-maintaining group (Group B). Specifically, patients whose GNRI score decreased by >10% from the preoperative period to the time of CGM measurement and/or whose nutritional risk category worsened were classified into the malnutrition-risk progression group (Group A), together with those whose GNRI category shifted to a higher-risk level. The remaining patients were assigned to the nutrition-maintaining group (Group B). We chose the >10% threshold because it not only corresponds to a one-category worsening in GNRI classification but also captures further nutritional deterioration in patients whose baseline GNRI was already <82. Because four patients were ≥1 year postoperative at the time of CGM (15–358 months), we defined the GNRI measured one year before CGM as the baseline for these cases. We then compared changes in plasma glucose levels, dietary intake, and body composition between the two groups.

### 2.3. Evaluation of Glycemic Management and Glucose Fluctuation by CGM

The average glucose level, time below range (TBR) (<70 mg/dL), TIR (70–180 mg/dL), and time above range (TAR) (>180 mg/dL) were calculated from the data obtained over three or more consecutive days while wearing the CGM device. We defined good glycemic targets for pancreatogenic diabetes as TIR > 50%, TAR < 50%, and TBR < 1% with reference to the recommendations from the international consensus [11].

### 2.4. Dietary Intake Surveys

Dietary surveys are routinely conducted by clinical nutritionists for patients with pancreatogenic diabetes mellitus after total pancreatectomy using home 3-day dietary recording and interview methods. Energy and nutrient intakes (carbohydrate, fat, and protein) were calculated from dietary surveys conducted from the postoperative period to the CGM measurement period.

### 2.5. Anthropometric Assessments

Weight, body fat percentage, body fat mass, skeletal muscle mass, and skeletal muscle index (SMI) were calculated using InBody770^®^ (InBody Inc., Tokyo, Japan) during outpatient visits or hospitalization at our facilities. SMI was calculated using bioelectrical impedance analysis (BIA).

### 2.6. Statistical Analyses

The Wilcoxon signed-rank test was used for comparing changes in GRNI between baseline and after total pancreatectomy using JMP Pro^®^ version 17.0.0 (SAS Institute, Cary, NC, USA). Comparisons between groups A and B were performed using the Mann–Whitney U test and Pearson’s chi-square test. The Mann–Whitney U test and Pearson’s chi-square test were conducted to compare groups A and B. Significance was set at *p* < 0.05.

In addition, we conducted univariable logistic regression analyses to examine the association between glycemic indices and malnutrition-risk progression. To address potential confounding, two-variable logistic regression models were fitted, including TIR and one covariate at a time (time since pancreatectomy, primary diagnosis, insulin regimen, or pancrelipase dose). We also constructed a model including TIR and pancrelipase dose per kilogram to specifically assess the potential effect of enzyme replacement therapy. We performed rank-based ANCOVA to evaluate group differences in TIR after adjusting for clinical covariates. Sensitivity analyses using binary TIR (>50% vs. ≤50%) with logistic regression models, each including one covariate at a time, were further carried out to confirm the robustness of the association.

## 3. Results

### 3.1. Patient Characteristics

The background and characteristics of the 14 patients are presented in Table 1. The median age was 70.0 years, and four were men. All patients were treated with pancrelipase as a pancreatic enzyme replacement, with a median daily dose of 1800 mg (IQR 1350–2250 mg), and five patients additionally received other digestive enzymes (Berizym^®^; Mucos Pharma GmbH & Co. KG, Berlin, Germany; distributed in Japan by Kyowa Pharmaceutical Industry Co., Ltd., Osaka, Japan), with daily dose of 6g. The primary indications for total pancreatectomy were pancreatic ductal adenocarcinoma (PDCA) (n = 6), intraductal papillary mucinous carcinoma (IPMC) (n = 5), pancreatic neuroendocrine tumor (PanNET) (n = 2), and pancreatic metastasis from renal cell carcinoma (n = 1). Thirteen patients were managed with multiple daily injections (MDI); one patient used CSII without a sensor-augmented regimen. Insulin dosing, including basal/bolus ratio, was titrated by the specialized physicians. CGM monitoring was performed during routine clinical care and generally contemporaneous with insulin adjustments, with a median duration of 10 days (IQR 6.8–16.5). The median time from pancreatectomy to initiation of CGM was 6 months (IQR 1–29). The median basal/total insulin ratio was 22.2% (IQR 14.2–28.8%); this likely reflects inclusion of early postoperative cases before stabilization of insulin requirements and the cautious reduction in basal insulin to minimize hypoglycemia.

**Figure 2:**
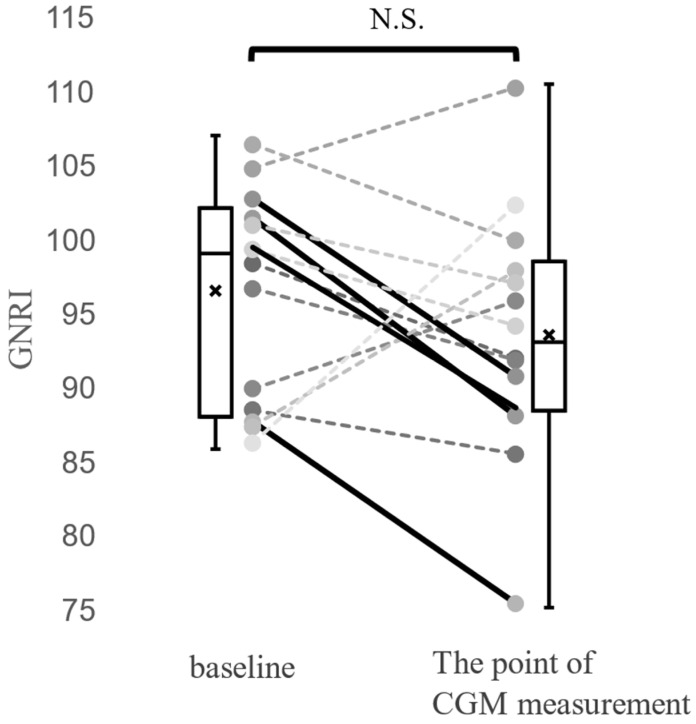
GRNI changes in the 14 patients with pancreatic diabetes mellitus after total pancreatectomy. Changes from baseline to the CGM measurement time point. Dotted line: GNRI decline ≤ −10%; solid line: GNRI decline >10%.CGM, continuous glucose monitoring; GNRI, geriatric nutritional risk index; N.S., not significant, *p* = 0.268 (Wilcoxon signed-rank test).

The median BMI, body fat percentage, and GNRI score were 19.5 kg/m^2^ (16.9–21.1 kg/m^2^), 18.2% (IQR 13.3–23.5%), and 92.5 (IQR 88.2–97.7), respectively. We observed 4 cases whose GNRI declined >10% from baseline to the time of CGM (Figure 2).

**Figure 3:**
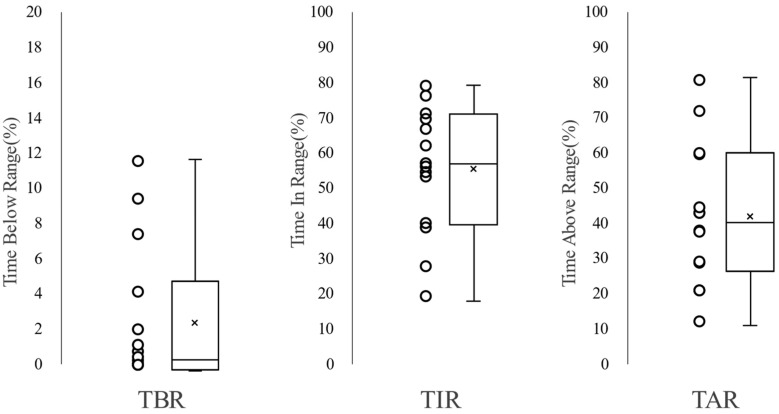
Distributions of time and range for each participant. Time below range (TBR): % reading and time < 70 mg/dL; time in range (TIR): % reading and time 70–180 mg/dL; and time above range (TAR): % reading and time > 180 mg/dL.

The overall glucose variability indices were HbA1c 7.3% (IQR 6.6–7.4%), mean glucose 166.3 mg/dL (IQR 152.7–194.1), TBR 0.41% (IQR 0.04–4.95%), TIR 63.4% (IQR 40.0–71.3%), TAR 36.2% (IQR 21.7–59.7%) (Figure 3).

The median daily energy intake was 35.6 kcal/IBW kg (IQR 31.4–41.7), although two patients consumed less than 25 kcal/kg.

### 3.2. Glycemic Comparison Between Malnutrition-Risk Progression (Group A) and Nutrition-Maintaining (Group B)

Patients were divided into groups A and B according to the criteria described in Section 2. Baseline characteristics, including gender, age, total insulin dose, or amount of pancreatic enzyme replacement, did not differ significantly between groups A and B (Table 1). No significant differences were observed in dietary intake (energy, protein, or fat), anthropometric parameters (BMI, lean body mass, body fat mass, and SMI), or nutritional parameters (Alb and T-Cho).

However, CGM analysis revealed that the mean glucose level and TAR were significantly higher, and TIR was significantly lower in group A compared with group B (Table 1). In addition, we calculated the glucose management indicator (GMI) from CGM-derived mean glucose values. The median GMI was 7.3% overall, 8.4% in group A, and 7.1% in group B. Similarly to mean glucose, GMI was significantly higher in group A compared with group B (*p* = 0.013). TIR and TAR, which achieved one of the clinical goals [11], were significantly higher in group B, although the TBR were comparable. Of the 14 patients, CGM detected that three with TBR > 5% during the day had greater TBR (11.7%, 11.8%, 14.3% at night.

Univariable logistic regression showed that lower TIR, higher TAR, and higher mean glucose were significantly associated with malnutrition-risk progression (Table 2). HbA1c, time since pancreatectomy, primary diagnosis, adjunct therapy, and pancrelipase dose were not significantly associated with these glycemic variables. To address potential confounding, we fitted additional two-variable models including TIR and one covariate at a time (time since pancreatectomy, primary diagnosis, or insulin regimen, Pancrelipase dose). Across these models, the inverse association remained consistent between TIR and malnutrition-risk progression (Table 3). For example, after adjustment for time since pancreatectomy, the odds of progression decreased by 65% per 10% increase in TIR (OR 0.35, 95% CI 0.07–0.82, *p* = 0.011). The effect of TIR was also robust to adjustment for primary diagnosis or insulin regimen. Furthermore, to specifically examine the potential confounding effect of pancreatic enzyme replacement therapy, we constructed an additional two-variable model including TIR and pancrelipase dose per kilogram (Table 3). Lower TIR remained significantly associated with malnutrition-risk progression (OR per 10% increase = 0.07, 95% CI 8.9 × 10^−6^–0.55, *p* = 0.0011). Although the pancrelipase dose appeared to be associated with progression (OR per 10 mg/kg = 51.4, 95% CI 1.25–2.7 × 10^7^, *p* = 0.033), the extremely wide confidence interval. indicated instability due to the small sample size. Taken together with the absence of group differences in reported dietary intake (Table 1), these findings support that the observed link between hyperglycemia and glycemic variability (lower TIR, higher TAR) and GNRI worsening was independent of dietary intake and enzyme replacement therapy.

To further account for potential confounders, we performed a rank-based ANCOVA for TIR between the groups (Supplementary Table S1). The group difference in TIR remained significant after adjusting for time since pancreatectomy, primary diagnosis, or pancrelipase dose. In sensitivity analyses using binary TIR (>50% vs. ≤50%), two-variable logistic regression models (including malnutrition-risk progression and one covariate at a time) also demonstrated a consistent inverse association between TIR and malnutrition-risk progression (Supplementary Table S2).

## 4. Discussion

In this study, we demonstrated that glycemic metrics may help identify patients at risk of malnutrition and guide postoperative management patients with pancreatogenic diabetes after total pancreatomy by continuous glucose monitoring (CGM).

The mean glucose level was significantly higher and TIR was significantly lower in the malnutrition-risk progression group (group A), whereas no significant differences were observed in other nutritional indices, dietary intake, or anthropometric assessments. Importantly, these associations remained consistent after adjustment for potential confounders in sensitivity analyses.

The GNRI was developed in 2005 by Bouillanne et al. [10] as a nutritional index calculated from ideal body weight, current body weight, and serum albumin levels to evaluate the risk of postoperative complications in older patients susceptible to malnutrition. Since then, GNRI has been shown to have prognostic value in various settings, including patients on hemodialysis [12] and those with heart failure [13,14]. In addition, lower GNRI scores have been associated with increased mortality and cardiovascular risk in older patients with diabetes [15], and GNRI has also been reported to predict the completion of adjuvant chemotherapy after surgery for PDAC [16,17]. Thus, GNRI has recently emerged as a useful prognostic marker in the fields of diabetes and pancreatic surgery as well. Nevertheless, to our knowledge, no previous studies have specifically examined the relationship between GNRI and glycemic variability after total pancreatectomy, which represents a novel aspect of the present investigation. Combining GNRI with CGM-derived metrics of glycemic variability may offer a more comprehensive assessment of postoperative risk and could ultimately contribute to improved outcomes in patients undergoing long-term management after total pancreatectomy. We specified a 10% reduction in GNRI as the definition of malnutrition-risk progression based on our observations. A previous study in peripheral artery disease indicated that a 10-unit decrease in GNRI was significantly associated with all-cause mortality, major adverse cardiovascular and leg events [18]. The result was consistent in our cohort when we defined the patients with a ≥10-unit decrease in GNRI as the malnutrition-risk group. This consistency supports the validity of our grouping method and indicates that our approach is in line with existing evidence.

In our study, basal/total insulin ratio was relatively low. This may reflect that our study included early postoperative cases before stabilization of insulin requirements. Recent evidence has shown that, although glycemic control and variability after total pancreatectomy are comparable to those in complete insulin-deficient type 1 diabetes, patients generally require lower total insulin doses. Moreover, insulin requirements after total pancreatectomy may vary by postoperative period, with higher needs in the immediate perioperative phase and lower long-term requirements compared with type 1 diabetes [19]. The association between lower TIR and worsening GNRI was consistent across sensitivity analyses, including a rank-based ANCOVA and a binary logistic regression (Supplementary Tables S1 and S2), further supporting the link between lower TIR and worsening nutritional risk. Importantly, the association between lower TIR and worsening GNRI remained robust even after adjustment for pancreatic enzyme replacement therapy (PERT) dose. Although PERT showed a possible association with nutritional risk (OR 1.89), the result was not statistically significant, and the wide confidence interval reflected the small sample size. Reported dietary intake also did not differ between groups. These findings suggest that glycemic variability, rather than enzyme dose or caloric intake, may play a central role in nutritional decline after total pancreatectomy. Shi et al. [20] reported that glycemic management after pancreatectomy affects early postoperative complications, with the complication rates (such as gastroparesis, abdominal bleeding, pneumonia, abdominal infection) being significantly higher when fasting glucose is high (>155 mg/dL), especially in the early postoperative period, and that postoperative HbA1c levels > 7% are associated with significantly shorter overall survival.

As a cause of malnutrition progression risk, hyperglycemia may have resulted in increased urinary glucose excretion and, therefore, loss of available energy. In Japanese patients, insufficient glycemic management is a risk factor for sarcopenia with type 1 and type 2 diabetes [21], and an increased HbA1c level is associated with a lean body mass reduction in type 1 diabetes [22]. In particular, Sugimoto et al. [23] reported that the frequency of sarcopenia increases with HbA1c levels in patients without obesity or type 2 diabetes. The detailed mechanisms underlying the detrimental effects of hyperglycemia on skeletal muscle are not fully understood, although several pathways have been postulated. The accumulation of advanced glycation end products is suspected to be one of the causes [24], as well as hyperglycemia-induced remodeling of the extracellular matrix of the skeletal muscle [25]. Moreover, a prolonged TAR may accelerate catabolism due to a lack of insulin action, thereby leading to a decrease in skeletal muscle mass. These previous findings support our results, suggesting that sustained hyperglycemia after total pancreatectomy may contribute to the loss of muscle mass and reductions in serum albumin, thereby promoting nutritional decline.

Nevertheless, an alternative explanation should also be considered. Patients with more advanced disease or greater frailty may have experienced deterioration in nutritional status primarily due to their underlying conditions. At the same time, such patients are often managed with less stringent glycemic targets, as recommended in international guidelines for frail populations [11]. Therefore, the observed association between nutritional risk progression and CGM metrics may not be explained by impaired metabolic control alone. It is also plausible that underlying disease severity and individualized treatment decisions might contribute to the observed differences.

In this study, we examined the association between CGM-derived indices and GNRI. CGM measures interstitial fluid glucose levels and translates them into blood glucose levels. Therefore, the absolute blood glucose values indicated by CGM may deviate from the actual measured blood glucose values. Deviations are especially likely to occur during blood glucose elevation and falls. Understanding trends in blood glucose fluctuations and the distribution of blood glucose zones is very useful; however, care must be taken when assessing local blood glucose levels at that point in time. A previous report showed that patients who underwent posttotal pancreatectomy had a higher TAR (>180 mg/dL) than those with type 1 diabetes, even for those with similar HbA1c levels [26]. These findings indicate that HbA1c alone is inadequate for assessing glycemic variability in postpancreatectomy patients, supporting the use of CGM for evaluation in this population. These results suggest that hyperglycemia correction may be necessary to prevent malnutrition in patients who undergo total pancreatectomy. In this context, CGM can contribute not only to the prevention of severe hypoglycemia but also to the early detection of hyperglycemia, which may help identify patients at risk of nutritional decline.

Although our study did not demonstrate a significant association between hypoglycemia and malnutrition progression risk, hypoglycemia remains a critical concern in postpancreatectomy patients. These patients lack both insulin and glucagon secretion, limiting their counter-regulatory capacity. Moreover, maldigestion, malabsorption, and depleted glycogen stores due to malnutrition may predispose them to prolonged hypoglycemia, particularly at night. CGM is valuable in identifying the timing of such events, enabling preventive strategies such as insulin adjustment or dietary modification [27]. Thus, beyond hyperglycemia, the role of CGM in detecting and preventing severe or unrecognized hypoglycemia should also be emphasized in this population.

This study has several strengths. First, to our knowledge, it is the first to demonstrate an association between TIR measured by CGM and nutritional risk assessed by GNRI in patients after total pancreatectomy. Second, we comprehensively evaluated the interplay between pancreatic endocrine and exocrine insufficiency, glycemic variability, and nutritional risk—an area that has been rarely investigated in this population. Third, the study provides practical implications, suggesting that CGM-based detection of glycemic abnormalities may offer opportunities for timely nutritional interventions and personalized management in clinical practice.

Concurrently, this study had several limitations. First, pancreatic exocrine insufficiency was not quantitatively assessed in this study population. We were unable to evaluate fecal elastase, coefficient of fat absorption, or fat-soluble vitamin levels in the retrospective dataset. However, none of the patients reported steatorrhea, and specified physicians optimally titrated pancreatic enzyme replacement therapy according to the patients’ condition. Second, CGM devices were not standardized, and both the monitoring duration and the time elapsed since pancreatectomy varied among the patients, which may have introduced heterogeneity in glucose indices. Third, this study may be considered preliminary due to its relatively small sample size in the two facilities, which reduces statistical power. Therefore, our results should be interpreted with caution. To overcome this limitation of the present study, we need to conduct a large prospective multicenter cohort study with single or standardized CGM device(s) and a fixed postoperative observation period in the near future.

## 5. Conclusions

In conclusion, our results suggest that hyperglycemia and glycemic fluctuation may be associated with malnutrition progression in patients with pancreatogenic diabetes mellitus after total pancreatectomy. CGM-based metrics, particularly TIR, may help flag patients at risk of nutritional decline, even when reported dietary intake and the enzyme replacement appear adequate. These findings are exploratory and will warrant confirmation in larger, prospective studies with standardized protocols.

## Figures and Tables

**Table 1:** Baseline characteristics of patients after total pancreatectomy.

	Overall (n = 14)	Group A (n = 4)	Group B (n = 10)	*p*-Value
Gender (male/female)	4/10	1/3	2/8	1.000
Age (years)	70.0 (61.0–76.0)	68.0 (57.8–73.0)	70.5 (59.0–76.3)	0.571
Primary diagnosis (PDAC/IPMC/PanNET/others)	6/5/2/1	1/2/0/1	5/3/2/0	0.171
Year of surgery	2017 (2014–2021)	2017 (2015–2020)	2017 (2014–2021)	0.943
Time since pancreatectomy (months)	6.0 (1.0–29.0)	1.0 (1.0–11.5)	8.0 (3.3–42.5)	0.198
Adjunct antidiabetic therapies, n (%)				
None	9 (64.3)	0	0	0.777
DPP-4 inhibitor	2 (14.3)	0	2 (20)	0.225
GLP-1 receptor agonist	1 (7.1)	1 (25)	0	0.100
Sulfonylurea	1 (7.1)	0	1 (10)	0.402
Glinide	2 (14.3)	1 (25)	0	0.100
Insulin dose				
Insulin regimen (MDI/pump)	13/1	4/0	9/1	-
Total insulin dose (U/day)	26.0 (20.0–30.3)	19.5 (15.1–28.0)	27.5 (23.4–31.8)	0.157
Insulin Basal/Total (%)	22.2 (14.2–28.8)	17.9 (14.8–22.9)	26.4 (13.9–30.1)	0.289
Digestive enzyme supplementation				
Pancrelipase (mg/day)	1800 (1800–2250)	1800 (1800–2137.5)	1800 (1800–2250)	1.000
Pancrelipase (mg/kg IBW)	40.0 (32.7–46.2)	46.8 (33.9–54.1)	38.4 (32.7–43.5)	0.289
Number (%) of other digestive enzyme concomitant users	35.7 (5/14)	25% (1/4)	40% (4/10)	0.728
Adherence *	No documented non-adherence in medical records	-	-	-
Steatorrhea	No patients reported steatorrhea during the observation period	-	-	-
Metabolic control				
HbA1c (%)	7.3 (6.6–7.4)	8.5 (6.3–9.2)	7.0 (6.7–7.3)	0.177
Average glucose (mg/dL)	166.3 (152.7–194.1)	213.2 (184.4–239.9)	159.4 (147.2–168.7)	0.013
Evaluation of glycemic management				
CGM device (rtCGM/ isCGM)	1/13	0/4	1/9	0.512
Duration of CGM use	10.0 (6.8–16.5)	10.0 (7.5–13.3)	11.0 (5.5–26.5)	0.943
GMI (%)	7.3 (7.0–8.0)	8.4 (7.7–9.1)	7.1 (6.8–7.3)	0.013
Time In Range (%)	63.4 (40.0–71.3)	34.0 (21.4–58.0)	65.6 (60.2–76.7)	0.040
Time Above Range (%)	36.2 (21.7–59.7)	65.7 (40.9–78.4)	32.4 (20.7–37.8)	0.048
Time Below Range (%)	0.41 (0.04–4.95)	0.25 (0.01–1.62)	0.74 (0.16–0.74)	0.567
Percentage of TIR target achieved (>50%)	10/14 (71%)	1/4 (25%)	9/10 (90%)	0.023
Percentage of TBR target achieved (<1%)	9/14 (64%)	3/4 (75%)	6/10 (60%)	0.728
Percentage of TAR target achieved (<50%)	10/14 (71%)	1/4 (25%)	9/10 (90%)	0.023
Achievement of 3 targets	5/14 (36%)	0/4 (0%)	5/10 (50%)	0.112
Achievement of 3 targets+HbA1c < 7.0	3/14 (21%)	0/4 (0%)	3/10 (30%)	0.414
Achievement of 3 targets+HbA1c < 7.5	5/14 (36%)	0/4 (0%)	5/10 (50%)	0.112
Dietary intake				
Energy (kcal/kg IBW)	34.6 (29.2–39.6)	33.8 (24.0–46.3)	34.6 (29.2–39.6)	1.000
Carbohydrate (g/day) (12 cases)	283.3 (250.3–315.3)	316.0 (276.0–332.0)	280.7 (214.1–300.2)	0.267
Protein (g/kg IBW) (12 cases)	1.4 (1.0–1.7)	1.39 (1.38–1.84)	1.36 (0.92–1.86)	0.579
Fat (g/day) (12 cases)	53.5 (47.3–67.6)	56.0 (47.0–87.0)	51.0 (40.5–65.2)	0.853
Anthropometric assessment				
Weight (kg)	45.8 (40.0–56.5)	39.8 (38.0–55.0)	47.2 (43.5–56.5)	0.288
BMI (kg/m^2^)	19.5 (16.9–21.1)	17.1 (15.9–22.9)	20.1 (18.0–22.9)	0.229
Body fat percentage (%) (10 cases)	18.2 (13.3–23.5)	11.5 (8.0–15.0)	19.5 (16.1–27.5)	0.090
Lean body weight (kg) (10 cases)	37.45 (31.5–50.5)	33.3 (32.2–34.3)	38.1 (35.2–47.7)	0.188
SMI (kg/m^2^) (8 cases)	5.9 (5.4–6.8)	5.2 (4.9–5.4)	6.6 (5.7–7.1)	0.094
Nutritional assessment				
Alb (g/dL)	3.9 (3.6–4.1)	3.6 (3.2–3.9)	4.0 (3.6–4.1)	0.086
T-Cho (mg/dL)	173.5 (160.3–200.0)	184.0 (113.0–210.8)	168.5 (160.3–200.0)	0.777
GNRI	92.5 (88.2–97.7)	88.0 (78.5–90.1)	96.5 (90.8–99.6)	0.020

Data are presented as median (IQR). rtCGM, real time CGM (Guardian™ Sensor 3^®^ [Medtronic Japan Co., Ltd., Tokyo, Japan]); isCG, intermittently scanned CGM (Freestyle Libre Flash Glucose Monitoring System^®^ [Abbott Japan LLC, Tokyo, Japan]); BMI, body mass index; SMI, skeletal muscle mass index; Alb, Albumin; T-Cho, total cholesterol; GNRI, geriatric nutritional risk index. * Adherence was evaluated retrospectively from medical records; “no documented non-adherence” indicates no record of poor adherence. No patients reported steatorrhea during the observation period.

**Table 2:** Univariable logistic regression for malnutrition-risk progression (n = 14).

Variable	OR	95% CI (Lower–Upper)	*p*-Value
TIR (per 10% increase)	0.34	0.15–0.76	0.008
TAR (per 10% increase)	2.88	1.28–11.68	0.006
Mean glucose (per 10 mg/dL)	2.22	1.22–8.43	0.003
HbA1c (per 1% increase)	2.64	0.68–19.3	0.165
Time since pancreatectomy (per year)	0.92	0.71–1.01	0.108
Primary diagnosis (PDAC vs. non-PDAC)	0.33	0.01–3.72	0.383
Pancrelipase dose (per 10 mg/kg)	1.89	0.51–8.69	0.334

OR = odds ratio; CI = confidence interval. *p*-values are from likelihood ratio tests.

**Table 3:** Logistic regression of TIR adjusted for potential confounders (n = 14).

Variables Included	OR for TIR (per 10% Increase)	95% CI (Lower–Upper)	*p*-Value (TIR)	OR for Covariate	95% CI (Lower–Upper)	*p*-Value (Covariate)
TIR only	0.34	0.15–0.76	0.008	–	–	–
TIR + Age	0.33	0.07–0.77	0.007	1.03	0.91–1.26	0.645
TIR + Time since pancreatectomy	0.35	0.07–0.82	0.011	0.88	0.56–1.02	0.161
TIR + Primary diagnosis (PDAC vs. non-PDAC)	0.25	0.012–0.75	0.006	0.11	0.0001–4.29	0.263
TIR + Insulin regimen (pump/automated vs. MDI)	0.35	0.085–0.81	0.01	0.00 (unstable)	<0.001–68.5	0.600
TIR + Pancrelipase dose (per 10 mg/kg)	0.07	8.9 × 10^−6^–0.55	0.001	51.4	1.25–2.7 × 10^7^	0.033

OR = odds ratio; CI = confidence interval. *p*-values are from likelihood ratio tests. The estimate for the insulin regimen was unstable due to very few pump/automated cases.

## Data Availability

The data that support the findings of this study are not publicly available due to privacy reasons, but are available from the corresponding author upon reasonable request.

## Supplementary Materials

The following supporting information can be downloaded at: https://www.mdpi.com/article/10.3390/jcm14197124/s1, Table S1: Rank-based ANCOVA of TIR between groups with and without malnutrition-risk progression; Table S2: Logistic regression using binary TIR (>50% vs ≤50%): sensitivity analyses.
