# Supplementary material for: CGM-Based Glycemic Metrics Support Estimating Nutritional Risk After Total Pancreatectomy: An Exploratory Retrospective Study
Source: J Clin Med. 2025 Oct 9;14(19):7124. doi: 10.3390/jcm14197124 (PMC12525270; doi:10.3390/jcm14197124)
Supplement: Supplementary file 1 [file jcm-14-07124-s001.zip › jcm-3888713-supplementary.pdf]

Supplementary Materials

Table S1. Rank-based ANCOVA of TIR between groups with and without malnutrition-risk progression.

Dependent Variable	Main Effect	Covariate (One at a Time)	Adjusted Difference in Ranks (Group A–B)	95% CI	*p*-Value
Rank_TIR	Group (A vs B)	None (unadjusted)	−5.25	−9.79 to −0.71	0.027
Rank_TIR	Group (A vs B)	Age	−5.18	−9.40 to −0.96	0.021
Rank_TIR	Group (A vs B)	Time since pancreatectomy	−6.05	−10.76 to −1.34	0.016
Rank_TIR	Group (A vs B)	Primary diagnosis (PDAC vs non-PDAC)	−5.69	−10.44 to −0.95	0.023
Rank_TIR	Group (A vs B)	Pancrelipase dose (mg/kg)	−6.17	−10.54 to −1.79	0.010

CI = confidence interval. Adjusted differences in ranks are shown for Group A (malnutrition-risk progression) vs Group B (nutrition-maintaining). p-values are from rank-based ANCOVA models adjusted for each covariate.

Table S2. Logistic regression using binary TIR (>50% vs ≤50%): sensitivity analyses.

Covariate Included	OR for TIR >50% (vs ≤50%)	95% CI	*p*-Value
None (unadjusted)	0.037	<0.01 to 0.57	0.017
Age(year)	0.036	<0.01 to 0.56	0.016
Time since pancreatectomy (months)	0.052	<0.01 to 0.89	0.041
Primary diagnosis (PDAC vs non-PDAC)	1.57×10^{-8}	<0.01 to 0.28	0.005
Pancrelipase dose (mg/kg)	0.0185	<0.01 to 0.45	0.011

OR = odds ratio; CI = confidence interval. p-values are from likelihood ratio tests. Each model included a binary TIR >50% and one covariate, due to the limited sample size.
